# Oronasal complications in patients after transsphenoidal hypophyseal surgery

**DOI:** 10.1016/S1808-8694(15)30649-2

**Published:** 2015-10-19

**Authors:** Carolina Petry, Carolina Garcia Soares Leães, Julia Fernanda Semmelmann Pereira-Lima, Katia D. Gerhardt, Geraldo Druck Sant, Miriam da Costa Oliveira

**Affiliations:** 1 Medical graduate, Ufcspa, Bolsista of the Centro de Neuroendocrinologia, Santa Casa de Porto Alegre/ UFCSPA; 2 Master's degree in Medicine: pathology; endocrinologist of the Centro de Neuroendocrinologia, Santa Casa de Porto Alegre/ UFCSPA; 3 Doutorate in Medicine; adjunct professor of internal medicine, UFCSPA; 4 Otorhinolaryngology resident, UFCSPA; 5 Doctorate in Otorhinolaryngology, adjunct professor of Otorhinolaryngology, UFCSPA; 6 Livre-Docente (habilitation) degree in Medicine, adjunct professor of endocrinology, UFCSPA

**Keywords:** pituitary diseases, hypophysectomy, pituitary gland

## Abstract

Transsphenoidal surgery is the most commonly used surgical procedure to handle the hypophyseal region, sometimes associated with oronasal complications.

**Material and methods/aim:**

To evaluate prospectively (specific questionnaire, clinical evaluation) undiagnosed chronic oronasal complications in patients submitted to conventional transsphenoidal adenomectomy surgery, operated at different neurosurgery services more than 6 months ago.

**Results:**

49 patients were evaluated, 37/45 presented macroadenoma. 28,5% were submitted to more than one intervention, 2/5 transsphenoidally. Transsphenoidal approach 92.8% through sublabial route. No patient had spontaneous complaint. With the specific questionnaire 63.2% presented complaints. One patient presented an oronasal fistula, 1 stenosis of the nasal valve area with external nasal deformity. Rhinoscopy detected alterations in 77.5%, nasal endoscopy in 87.7%. Septal perforation was present in 10/12 patients with scabs and 2 with purulent secretion. All 4 patients submitted to 2 transsphenoidal approaches presented septal perforation and nasal synechiae. In the endonasal, synechiae (2), alteration in medium meatus (1) and stenosis of the nasal valve area (1) were observed. Only two patients presented normal evaluation.

**Conclusion:**

A high incidence of nasal complications after conventional transsphenoidal surgery observed through examination and not reported spontaneously point to the need of otorhinolaryngological investigation complemented by nasal endoscopy in patients submitted to procedures through this route.

## INTRODUCTION

Hypophyseal gland conditions have been a concern for neurosurgeons and otorhinolaryngologists since the late 19th century; both specialties have helped develop surgery of the sellar area. Transsphenoidal surgery is the most commonly used procedure to approach the hypophyseal region.

Intranasal transsphenoidal access to the sella turcica for removing pituitary gland tumors has been known since the beginning of the last century; it was developed because the beginning of intracranial surgery for hypophyseal tumors was difficult. Techniques involved major surgery, done by a superior nasal approach; it was traumatic, including manipulation of many paranasal sinuses, which increased the risk of postoperative infection. Access was changed to the infranasal or sublabial approaches, which avoided having to enter the ethmoidal cells, thus reducing the risk of infection. Halsted^1^ was the first to use a sublabial, rather than an infranasal, incision; this was the last step in the development of a relatively safe and direct approach through the midline to the hypophysis. Hardy^2^,^3^ popularized the technique, to which was added the surgical microscope and fluoroscopy; it has become the procedure of choice for the surgical treatment of pituitary lesions.

Although safe and effective, transnasal microsurgery of the hypophysis has at times been associated with oronasal complications that affect the sinuses, bone and cartilaginous structures, and teeth. Nasal complications predominate, at a frequency of up to 38% (obstruction and crusting) after the procedure.^3^,^4^ These findings appear to favor the nasal endoscopic approach, which has been associated with fewer local complications.^5^, ^6^, ^7^

The purpose of this study was to assess the frequency of undiagnosed chronic oronasal alterations in a series comprising 49 patients undergoing conventional transsphenoidal surgery.

## SERIES AND METHOD

The study sample comprised 49 patients of a neuroendocrinology outpatient unit that had previously undergone adenomectomy by a hypophyseal transsphenoidal approach; these patients were operated in different neurosurgery units. There were 17 male and 32 female patients aged from 30 to 72 years.

An inclusion criterion was a more than six-month interval between surgery and entry into the study. Patients answered a specific questionnaire for oronasal cavity signs and symptoms and were examined medically by a single otorhinolaryngologist to identify possible oronasal alterations; the examination consisted of nasal inspection, anterior rhinoscopy and nasal rigid 30o endoscopy with 10% lidocaine nasal spray anesthesia. Patient file data were gathered on the hormonal phenotype of the pituitary adenoma, its anatomical features in the pre-surgical sellar image, and computed tomography of the facial sinuses after surgery, if available.

Patients consented to participate in the study, which the Research Ethics Committee of the institution approved (n° 868/04).

## RESULTS

### Sample

Fourteen (28.5%) of 49 patients had undergone more than one hypophyseal intervention; the transsphenoidal approach was used in both procedures in five patients.

In 37/45 patients (75.5%) preoperative image data showed that the tumor was a pituitary macroadenoma. Phenotypically, adenomas were clinically non-functioning in 24 cases (48.97%), associated with acromegaly in 16 cases (32.6%), associated with Cushing's disease in 5 cases (10.2%), and with prolactin hypersecretion in the remaining cases (8.16%).

The transsphenoidal approach, which was known in 42 cases, was sublabial in 39 cases (92.8%) and endonasal transeptal in three cases. The interval between surgery (or in transsphenoidal reoperation cases of the last surgery) and the rhinological examination ranged from 7 months to 24.7 years. No patient underwent otorhinolaryngological surgery after the hypophyseal intervention.

### Clinical manifestations

At no time in consultation were there any spontaneous complaints of surgically related nasal or oral symptoms, headaches, facial pain or other possible complications. In the specific questionnaire, 31 patients (63.2%) presented postoperative complaints (Table 1). Nine, eight, six, four, two, one and one subjects presented respectively from one to seven complaints. Table 2 shows the number of complaints related with the hormonal feature of adenomas.

**Table 1:** Complaints pertaining to oronasal complications in patients undergoing transsphenoidal surgery.

	Complaint	n (%)
	Crusting	14 (28)
	Obstruction	12 (24)
	Irritation	11 (22)
Nose	Hyposmia/Anosmia	10 (20)
	Secretion of pus*	6 (12)
	Recurring epistaxis	4 (8)
	Cacosmia	3 (6)
	Anesthesia	2 (4)
	Lip anesthesia	4 (8)
Mouth	Altered dental sensitivity	3 (6)
	Gingival anesthesia	2 (4)
	Decreased sense of taste	1 (2)
	Headache	6 (12)
Others	Facial pain	3 (6)
	Epiphore	1 (2)

Lasting from 2 weeks to 7 years.

**Table 2:** Frequency of complaints and alterations in the otorhinolaryngological exam in patients undergoing transsphenoidal surgery for the treatment of pituitary adenoma

Diagnosis	Complaints	Number of alterations in exam
Non-functioning adenomas (n=24)	(0 a 4)	(0 a 5)
Acromegaly (n=16)	(0 a 7)	(0 a 5)
Cushing's disease (n=5)	(0 a 4)	(1 a 4)
Prolactinoma (n=4)	(0 a 5)	(1 a 2)
Total number of secretors (n=25)	(0 a 7)	(0 a 5)

In patients reporting facial pain the oronasal examination revealed no secretion of pus.

### Oronasal examination

Oroscopy and the external nasal inspection revealed alterations in two patients: one had an oronasal fistula and the other presented right nasal valve stenosis with external nasal deformity. Anterior rhinoscopy detected alterations in 38 patients (77.5%); nasal endoscopy revealed findings in 43 patients (87.7%) (Table 3). One to five alterations were detected in 12, 16, eight, five and three subjects respectively.

**Table 3:** Anterior rhinoscopic and nasal endoscopic findings in patients undergoing transsphenoidal surgery.

Alteration	Rhinoscopy n (%)	Endoscopy n (%)
Septal perforation	29 (59)	30 (61)
Synechiae*	18 (36)	24 (48)
Crusting	12 (24)	12 (24)
Secretion of pus	1 (2)	2 (4)
Nasal valve stenosis	1 (2)	–
Middle meatus alterations**	–	4 (8)
Sphenoid recess alterations***	–	3 (6)

* Between the septum and turbinates (inferior or middle) in one or both nasal fossae

** Block, edema

*** Wide sinusotomy in two cases; significant edema and thick mucous secretion in another case

Septal perforation was found in 10 of 12 patients with skin crusting and in the two patients with secretion of pus seen in anterior rhinoscopy.

Complaints/findings in the examination were 1/4, 5/5, 0/4 and 6/4 in the four patients that underwent two transsphenoidal procedures. All presented septal perforation and nasal synechiae.

Synechiae were found in two of the three cases in which an endonasal approach was used; additional findings in these three patients were middle meatus alterations (block, edema)^1^ and right nasal valve stenosis.^1^ Among the entire sample, 10 postoperative facial sinus computed tomographies were found. Changes were found in 8 exams: septal perforation (5 cases), sphenoid filling/opacification,^1^ sphenoid and ethmoidal filling/opacificaiton,^1^ sphenoid sinus mucosal thickening,^4^ sphenoid and maxillary sinus mucosal thickening,^1^ osteítis,^1^ posterior ethmoidal sinusitis,^1^ and pansinusitis.^1^

The evaluation was normal in only two patients: these patients had no complaints, and no alterations in oroscopy/rhinoscopy or in facial sinus computed tomography.

## DISCUSSION

The transsphenoidal approach is a safe and effective surgical procedure; the mortality rate in most series ranges from 0 to 1%,8 although at times there are mainly endocrinological complications. Oronasal complications due to this procedure may include the facial sinuses, bone and cartilaginous structures, and teeth.

A number of complaints and rhinoscopic/endoscopic alterations were found in our series. The most frequent complaints were crusting, nasal obstruction and irritation, and altered olfaction. The percentage of nasal obstruction and nasal crusting was a little below the reported average in the literature: 38% in Monnier's series evaluating the transvestibular transeptal approach.^4^ On the other hand, chronic nasal irritation - found in nearly one fifth of our patients - was seen in only 2% of cases in Feigenbaun et al.'s^9^ series. The occurrence of the most frequent nasal endoscopic finding in our series, septal perforation, was significantly higher than Dew's^10^ reported 18%.

Sinusitis was similar to other reported percentages (1%,^11^ 3%^9^ and 12%^12^); labial, gingival or dental dysestesias were reported in 13 and 37% of cases.^10^

Such variability in findings in due to a number of factors, including the surgical approach and the experience of the surgical team.^13^,^14^ Comparisons between the sublabial and endonasal transeptal approaches have yielded varying results. While some authors have reported no differences between these techniques,^15^ others have shown that there are fewer complications in the endonasal approach;^16^,^17^ these authors argue for the transnasal approach, in which the access route is shorter and more directs, notwithstanding a smaller operative field.^18^ Yamada^19^ proposed a modified sublabial approach with fewer mucosal complications compared to the conventional approach. We did not compare the results of different approaches because the number of endonasal approaches in our series was small.

Postoperative results of endonasal endoscopy have been published since 1994.^20^, ^21^, ^22^ A comparison between the endonasal endoscopy and the sublabial approach has revealed that both are equally effective; in endoscopy, however, the hospital stay, duration of surgery and complication rates were lower.^20^ According to these authors, patients undergoing endoscopic surgery had fewer complications compared to patients operated with the sublabial approach (4.5% vs. 27%). White et al. also found a significant reduction in the occurrence of epistaxis, lip anesthesia and septal deviation.^7^

## CONCLUSION

This study confirms the high incidence of nasal complications following hypophyseal surgery by the conventional transsphenoidal approach, seen in a specialized examination, albeit not necessarily reported spontaneously by patients. These findings suggest that otorhinolaryngological investigation with nasal endoscopy and facial sinus computed tomography is required in patients operated by this approach.
